# Efficacy and Safety of Dupilumab in Chinese Patients With Atopic Dermatitis: A Real-World Study

**DOI:** 10.3389/fmed.2022.838030

**Published:** 2022-03-23

**Authors:** Bingjing Zhou, Cong Peng, Liqiao Li, Runqiu Liu, Lei Zhu, Xiang Chen, Jie Li

**Affiliations:** ^1^Department of Dermatology, Xiangya Hospital, Central South University, Changsha, China; ^2^Hunan Key Laboratory of Skin Cancer and Psoriasis, Xiangya Hospital, Central South University, Changsha, China; ^3^National Clinical Research Center for Geriatric Disorders, Xiangya Hospital, Central South University, Changsha, China

**Keywords:** atopic dermatitis, biological agents, dupilumab, efficacy, real world

## Abstract

**Background:**

Atopic dermatitis (AD) is a common skin disease, but treatment of this disease has been challenging. Dupilumab is a new biological agent for AD that has been proven to be safe and effective in clinical trials. Although dupilumab was approved for listing in China in June 2020, real-world data about the application of dupilumab in China are lacking. This study aimed to collect and analyze real-world data on dupilumab among Chinese AD patients.

**Methods:**

Demographic and clinical data for 116 AD patients receiving dupilumab treatment were reviewed. The Eczema Area and Severity Index (EASI), SCORing Atopic Dermatitis (SCORAD), Numerical Rating Scale (NRS), Patient Oriented Eczema Measure (POEM), and Dermatology Quality of Life Index (DLQI) of patients were evaluated every 2 weeks from baseline to 16 weeks of treatment. Any adverse events during treatment were recorded.

**Results:**

Among the 116 patients in this study, baseline levels of IgE, eosinophils, and LDH were elevated in 62.79% (*n* = 86), 45.30% (*n* = 86), and 54.20% of patients (*n* = 48), respectively. The SCORAD index and POEM, DLQI, and NRS scores were significantly improved in all patients at 2 weeks (*p* < 0.0001), 4 weeks (*p* < 0.01), and 16 weeks (*p* < 0.001). EASI scores also improved significantly in all patients at 2 weeks (*p* < 0.01), 4 weeks (> 0.05), and 16 weeks (*p* < 0.01). However, 11 patients (9.48%) had no response. IgE and LDH levels (*p* > 0.05), Eosinophil counts (*p* < 0.01) in blood increased temporarily in the first 4 weeks and then decreased and stabilized during dupilumab treatment. Conjunctivitis was the most common adverse event (2.59%) among the patients. We found that the curative efficacy of dupilumab at 4th weeks was related to the patient’s age and course of disease. Nevertheless, there is no relationship between levels of eosinophils, IgE, LDH and the therapeutic efficacy of dupilumab.

**Conclusion:**

The real-world data in China showed that dupilumab can effectively treat AD and is well tolerated with a low incidence of adverse events.

## Introduction

Atopic dermatitis (AD) is an inflammatory, chronically recurrent skin disease that is more likely to occur in people with a family history of allergies (1). Recent studies reported that Timothy, birch and house dust mite were the main allergens in AD patients, whereby molecular components including Phl p 1 (Timothy), Bet v 1 (Birch), Alt a 1 (*Alternaria*) play a significant role in the atopic march (2, 3). AD usually occurs in early childhood but can develop at any age (4). The incidence rate of AD has increased globally over the years (5). Guo et al. (6) found a prevalence of AD in Chinese children aged 1–7 years of 12.94%, higher than in previous reports. From a pathological point of view, AD manifests as thickening of the epidermis, infiltration of dendritic cells and T lymphocytes, and increases in inflammatory mediators (7, 8). The number of inflammatory factors, such as IL-4, IL-13, and IL-31, produced by Th2 cells is significantly increased in the skin of patients with AD, suggesting that AD is mainly driven by type 2 inflammation (9, 10). These data indicate that targeted therapy for Th2 inflammation may help to alleviate AD.

Dupilumab is a fully human anti-IL-4 receptor α (IL-4α) monoclonal antibody that blocks signaling mediated by IL-4 and IL-13 (11, 12) and the first biological agent used for AD. The results of clinical trials at 16 weeks and 52 weeks in patients with moderate to severe AD showed that dupilumab significantly improved the disease in the extension of visible lesions, itch intensity, pain and impact of skin disease on daily activities, work and social life by inhibiting inflammatory cell and the type 2 inflammatory response (13–15). Accumulating evidence supports the conclusions that the eczema area and severity index (EASI), SCORing Atopic Dermatitis (SCORAD), Numerical Rating Scale (NRS), Patient Oriented Eczema Measure (POEM), and Dermatology Quality of Life Index (DLQI) were the most widely used tools to evaluate treatments in AD. Treatment points in AD with dupilumab focus on clinician-reported disease severity, patient-reported symptoms, and impact on quality of life and long-term control. Recent studies supported that after dupilumab treatment, patients who achieved clinical improvement in at least one of primary (EASI 75) or secondary (NRS peak pruritus improvement ≥4 or DLQI improvement ≥4) endpoints were considered to have experienced good therapeutic effects (16–18). Additionally, dupilumab has an acceptable safety profile, and does not require laboratory monitoring during the working period (19).

There are limited data from Asia about existing real-world uses of dupilumab. Thus, for the purpose of researching the effectiveness and safety of dupilumab in the real world in China, we report actual data for dupilumab in the treatment of AD.

## Materials and Methods

All patients with AD treated with dupilumab from the Department of Dermatology, Xiangya Hospital, Central South University between August 2020 and November 2021 were included, and all the data were collected from patient medical records and questionnaires. AD was diagnosed by two dermatologists independently according to the revised Hanifin and Rajka criteria (20). This research was approved by the Medical Ethics Committee of Xiangya Hospital, Central South University and conducted in accordance with research ethics (Ethical approval number: 201904112).

Patients (body weight more than 30 kg) were subcutaneously injected with 600 mg dupilumab for the initial dose and 300 mg dupilumab for the maintenance dose. For children with body weights less than 30 kg, the first dose and maintenance dose were 300 mg once every 3 weeks. Usually, the interval time between the two subcutaneous injections in adults was 2 weeks and adjusted according to the SCORAD after 16 weeks of treatment. Due to economic and distance reasons, the interval of medication was 3 or 4 weeks for 14 patients (12.07%). According to the evaluation of dermatologists, after the patient’s condition was significantly improved, whereby EASI improved by more than 50% from baseline or DLQI, NRS, and POEM scores improved by at least 4 points from baseline, the interval was extended from 2 weeks to 3 or 4 weeks. Three patients (23.08%) received dupilumab continuously up to 16 weeks were not treated with the classical 2-week interval during the whole treatment process. After a course of treatment or after the patient has reached the end point of treatment, it is changed to topical medication.

As indicative factors for AD, EASI, SCORAD, NRS, POEM, and DLQI were measured before treatment and at 2, 4, or 16 weeks after the first dose of dupilumab (21). In addition, serum IgE levels, serum C-reactive protein levels, lactate dehydrogenase (LDH) levels and eosinophil counts were measured, and electrocardiograms and chest radiography were used to help exclude potential comorbidities at enrollment.

### Statistical Analyses

Binary logistic regression was used to analyze the correlation between patient characteristics (age, sex, allergy history), baseline eosinophil counts, LDH and IgE levels and outcomes. The Kruskal–Wallis signed-rank test was applied to analyze the changes in eosinophil, IgE, and LDH levels and EASI, SCORAD, POEM, DLQI, and NRS scores at baseline and 2, 4, or 16 weeks after the first administration of dupilumab. Statistical significance was defined as *P* < 0.05. All analyses were performed with the Social Sciences Statistical Package (version 24.0, SPSS Inc., Chicago, IL, United States).

## Results

### Baseline Characteristics of Atopic Dermatitis Patients

One hundred sixteen Chinese patients (81 males and 35 females) were recruited for this study. Their mean age was 35.91 (±23.81) years. The duration of AD was 6.52 (±5.19) years. The demographics, medical history (including family history), atopic/allergic diseases at baseline (i.e., allergic rhinitis, allergic asthma, etc.), accompanying medications, adverse events, and treatment responses of patients were recorded. Table 1 is a brief summary of the demographics and clinical characteristics of the patients. The therapeutic efficacy of dupilumab at 4th weeks was related to the patient’s age and course of disease (*p* < 0.05) but not sex, atopic/allergic diseases at baseline (*p* > 0.05) (Table 2).

**TABLE 1:** Baseline characteristics of AD patients included in this study (*N* = 116).

Characteristics	Mean (±SD) or Count (%)
Age (year)	35.91 (±23.81)
Gender	Male, 81 (69.83%); female, 35 (30.17%)
Duration of AD (year)	6.52 (±5.19)
Family history of allergy	10 (8.62%)
Atopic/allergic diseases at baseline	44 (37.93%)
Allergic rhinitis	30 (25.86%)
Asthma	10 (8.62%)
Allergic conjunctivitis	4 (3.45%)
**History of previous topical treatments**	
Topical corticosteroids	116 (100%)
Halometasone	38 (32.76%)
Mometasone	34 (29.31%)
Desonide	19 (16.38%)
Triamcinolone	4 (3.45%)
Topical calcineurin inhibitors	19 (16.38%)
Tacrolimus	14 (12.07%)
Pimecrolimus	5 (4.31%)
**History of previous systemic treatments**	
Antihistamines	116 (100%)
Lupatadine	14 (12.07%)
Levocetirizine	27 (23.28%)
Loratadine	29 (25.00%)
Bepotastine	15 (12.93%)
Ebastine	12 (10.34%)
Systemic immunosuppressants	13 (11.21%)
Cyclosporine	7 (6.03%)
*Tripterygium wilfordii*	6 (5.17%)
Systemic glucocorticoids	5 (4.31%)
Prednisone	3 (2.59%)
Dexamethasone	2 (1.72%)
**Concomitant topical medications**	
Halometasone	37 (31.90%)
Desonide	19 (16.38%)
Mometasone	25 (21.55%)
Loratadine	18 (15.52%)
Levocetirizine	21 (18.10%)
Bepotastine	24 (20.69%)
**Comorbidities**	
Chronic kidney dysfunction	7 (6.03%)
Chronic hepatitis B	4 (3.45%)
Diabetes	4 (3.45%)
Serum IgE level (IU/ml) (*N* = 86)	2000.00 (±4670.00)
Number of circulating eosinophils (×10^9^/L) (*N* = 86)	0.76 (± 0.67)

**TABLE 2:** The relationship between clinical characteristics and efficacy at 4 weeks (EASI improvement of more than 50% at 4 weeks is defined as effective, of which 33.33% is effective) (*N* = 27).

Characteristics	Mean (±SD) or Count (%)	OR	95% CI	*P*-value*
Male	Effective: 6 (66.67%) Non-effective: 10 (55.56%)	21.198	0.241–1867.144	0.181
Age (year)	Effective: 43.00 (±17.96) Non-effective: 19.72 (±13.30)	1.216	1.035–1.501	0.020
Course of disease (year)	Effective: 8.81 (±8.34) Non-effective: 5.25 (±3.96)	1.491	1.019–2.181	0.040
Atopic/allergic diseases at baseline	Effective: 2 (22.22%) Non-effective: 10 (55.56%)	6.586	0.097–447.959	0.381
Baseline serum IgE level (IU/ml) (*N* = 23)	Effective: 454.40 (±570.80) Non-effective: 1572.00 (±1963.00)	0.999	0.997–1.001	0.174
Baseline eosinophil counts (×10^9^/L) (*N* = 24)	Effective: 0.97 (±0.62) Non-effective: 0.80 (±0.69)	1.375	0.373–5.071	0.633
Baseline serum LDH level (U/L) (*N* = 13)	Effective: 256.30 (±57.37) Non-effective: 339.9 (±123.80)	0.991	0.976–1.006	0.226

*P-value was calculated by Binary logistic regression.

Prior to dupilumab treatment, patients had received multiple medications (topical glucocorticoids and/or topical calcineurin inhibitors, systemic glucocorticoids, antihistamines, *Tripterygium wilfordii*, immunosuppressants). All patients had received topical glucocorticoids and antihistamines but responded poorly. The patient’s previous medication history for AD is described in Table 1. Seventy-seven patients (66.38%) were subsequently treated with dupilumab in combination with topical glucocorticoids or topical calcineurin inhibitors because they were used before dupilumab treatment. All patients were advised to implement skin moisturizing care every day.

### Eczema Area and Severity Index, SCORing Atopic Dermatitis, Patient Oriented Eczema Measure, Dermatology Quality of Life Index, Numerical Rating Scale Scores

The mean baseline EASI score was 19.47 (±16.07). Among the patients with at least 0, 2, 4, and 16 weeks of follow-up (*N* = 116), 50.00, 22.41, and 11.21% received dupilumab continuously up to 2, 4, and 16 weeks, respectively. As mentioned above, the primary (EASI 75) or secondary (NRS peak pruritus improvement ≥4 or DLQI improvement ≥4) endpoints were concerned during treatment (17). After dupilumab treatment, 102 patients (87.93%) showed significant improvement in skin rash. In 103 patients (88.79%) who had not used dupilumab for 16 weeks, 61 (59.22%) were evaluated as reaching the end point of efficacy. Changes in the mentioned evaluation criteria are given in Table 3.

**TABLE 3:** Scoring characteristics in patients with AD at baseline and follow-up after 2, 4, and 16 weeks.

	Baseline (*N* = 102)	Week 2 (*N* = 58)	*P*-value*	Week 4 (*N* = 26)	*P*-value*	Week 16 (*N* = 13)	*P*-value*
EASI score	19.47 (±16.07)	11.49 (±9.13) (*N* = 57)	0.0028	13.94 (±13.60)	0.1812	5.43 (±7.29)	0.0016
SCORAD index	56.28 (±15.73) (*N* = 99)	39.28 (±12.14) (*N* = 53)	<0.0001	39.39 (±19.15) (*N* = 24)	<0.0001	26.47 (±14.51) (*N* = 11)	<0.0001
POEM score	19.81 (±5.41) (*N* = 101)	10.62 (±4.92)	<0.0001	9.92 (±5.93)	<0.0001	8.62 (±5.77)	<0.0001
DLQI score	14.28 (±7.24) (*N* = 101)	7.53 (±4.23) (*N* = 57)	<0.0001	9.42 (±6.33)	0.0014	6.08 (±3.80) (*N* = 12)	0.0006
NRS score	8.06 (±2.32)	4.85 (±2.09)	<0.0001	4.08 (±2.19)	<0.0001	3.77 (±2.46)	<0.0001

*P-value was calculated by Kruskal–Wallis signed-rank test.

At 2 weeks, the decrease was 30.36% in the SCORAD index, 46.39% in the POEM, 47.27% in the DLQI, 43.80% in the NRS (*p* < 0.0001), and 40.99% in the EASI (*p* < 0.01). At 16 weeks, all scoring index had decreased significantly, with the SCORAD index by 52.97%, POEM by 56.49% and NRS by 53.23% (*p* < 0.0001), DLQI by 57.42% (*p* < 0.001), and EASI by 72.11% (*p* < 0.01) (Table 3).

**FIGURE 1:**
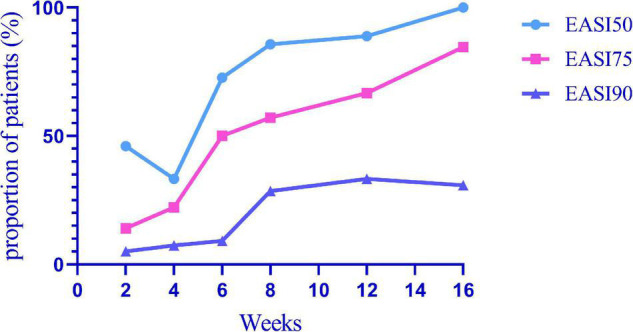
EASI 50, EASI 75, and EASI 90 indicate the proportion of patients with an improvement over 50, 75, and 90% in EASI from 2 to 16 weeks after the first subcutaneous dupilumab.

EASI 50, EASI 75, and EASI 90 indicate the proportion of patients with improvement over 50, 75, and 90% based on the EASI from 2 weeks to 16 weeks (Figure 1). Among all patients, EASI 50, EASI 75, and EASI 90 were 46.00, 14.00, and 5.00% (*n* = 57), respectively, 2 weeks after the first subcutaneous injection of dupilumab. At 16 weeks, EASI 50, EASI 75, and EASI 90 were 100, 84.62, and 30.77%, respectively (*n* = 13).

### Laboratory Tests

The baseline serum IgE level was increased in 54 of 86 patients (62.79%). There was no apparent decline in serum IgE levels after the first administration of dupilumab, and there was no significant difference after 16 weeks (*p* > 0.05) (Table 4). Two patients even exhibited an increased serum IgE level during treatment; in one of them, it increased from 1433.3 to 8683.7 IU/ml. Nevertheless, their clinical symptoms improved. After the second dose of dupilumab, one patient had an improvement in the EASI of approximately 75%, suggesting that the improvement in AD symptoms did not correlate with the level of IgE (Table 2).

**TABLE 4:** Laboratory test in patients with AD at baseline and follow-up after 2, 4, and 16 weeks.

	Baseline (*N* = 86)	Week 2	*P*-value*	Week 4	*P*-value*	Week 16 (*N* = 8)	*P*-value*
Serum IgE level (IU/ml)	2000.00 (±4670.00)	3067.00 (±3614.00) (*N* = 7)	0.9812	4735.00 (±5878.00) (*N* = 5)	0.6555	964.20 (±1548.00)	0.9784
Eosinophil count (×10^9^/L)	0.76 (±0.67)	1.62 (±1.89) (*N* = 11)	0.0078	2.98 (±1.30) (*N* = 4)	<0.0001	0.55 (±0.48)	0.8890
Serum LDH level (U/L)	304.10 (±128.90) (*N* = 48)	371.10 (±284.90) (*N* = 4)	0.6035	/	/	259.00 (*N* = 1)	0.9402

*P-value was calculated by Kruskal–Wallis signed-rank test.

According to a real-world study from South Korea, LDH was significantly decreased at 16 weeks, and patients with LDH value ≥250 U/L at 16 weeks showed a significantly smaller change in NRS, POEM, and DLQI from baseline, but LDH levels at baseline were not related to 16-week efficacy (22); Similarly, LDH levels at baseline were not associated with the efficacy of dupilumab in our study (Table 2). There was no difference in LDH levels before and after treatment (Table 4), which may be due to the small sample size of our patients. Large samples are needed in the future to find biomarkers for predicting the efficacy of dupilumab.

Eosinophil counts were elevated in the peripheral blood of 45.30% of patients (*n* = 39/86). Eosinophil counts in blood increased temporarily (*p* < 0.01) in the first 4 weeks and then decreased and stabilized during dupilumab treatment. One patient’s eosinophil numbers continued to increase from 2.48 × 10^9^/L to 3.14 × 10^9^/L, with poor efficacy, during treatment. The study in South Korea also mentioned that patients with higher eosinophil levels at baseline or at 16 weeks had a poorer response to dupilumab treatment (22). We did not find this correlation because of the relatively small sample size (Table 2).

### Safety

**FIGURE 2:**
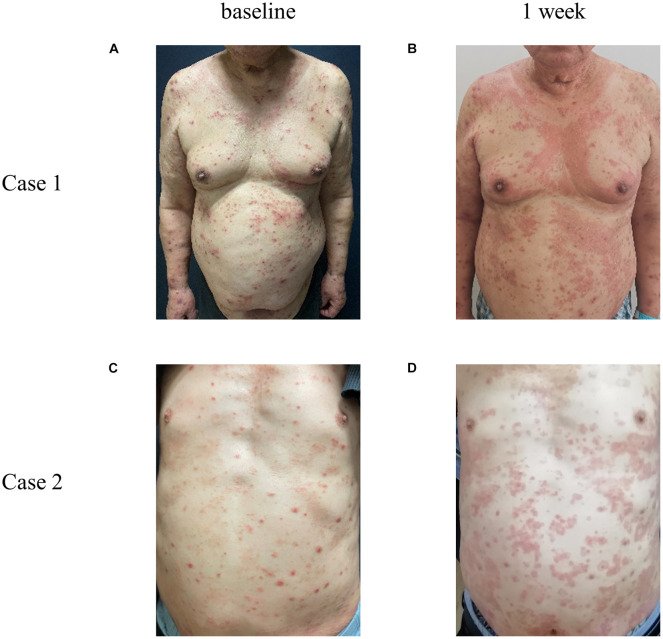
Representative photos of AD patients with allergic reaction after subcutaneous injection of dupilumab. Case 1 and 2 represent two different patients. Compared with the condition at baseline **(A,C)**, 1 week **(B,D)** after the first subcutaneous injection of dupilumab, a large number of edematous erythema and papules suddenly appeared all over the body, fused into a piece, with fine scales on the surface, accompanied by severe itching.

With regard to the safety of dupilumab, seven patients (6.03%) had adverse events: three patients developed conjunctivitis during treatment, and they all had a history of conjunctivitis. Conjunctivitis improved after using tobramycin eye drops and levofloxacin eye drops. One of the patients stopped using dupilumab as a result of poor efficacy and side effects. One patient had transient oral herpes simplex and received valaciclovir treatment; after that, she recovered without recurrence according to follow-up, indicating that dupilumab does not increase the risk of herpes simplex. Three patients had adverse drug reactions, one of whom presented with an injection site reaction such as transient erythema and improved on its own; the other two had extensive drug eruption the day after receiving subcutaneous injection of dupilumab (Figure 2). They were treated with dexamethasone and antihistamines in combination with TCS. After 7 days of treatment, their eruption was obviously alleviated, and other treatments were used.

## Discussion

**FIGURE 3:**
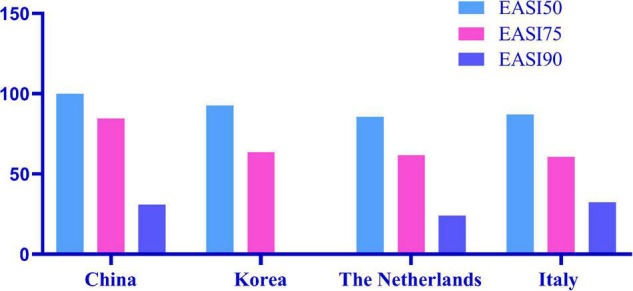
Real world data on EASI 50, EASI 75, and EASI 90 from different countries.

Among the 116 patients with AD, 102 had significant improvement in clinical manifestations, even some patients were not treated with the classical 2-week interval. Based on the SCORAD and EASI scores at the 2nd, 4th, and 16th weeks, we found that the disease severity of the patients decreased significantly. According to the NRS, itching was basically controlled, and the DLQI score showed that the quality of life of patients had been greatly improved. In our study, among all patients, EASI 50, EASI 75, and EASI 90 were 46.00, 14.00, and 5.00%, respectively, at 2 weeks after the first subcutaneous dupilumab. At 16 weeks, EASI 50, EASI 75, and EASI 90 were 100.00, 84.62, and 30.77%, respectively. In a Korean cohort study, the proportions of EASI 50 and EASI 75 were 51.4 and 2.8% at 2 weeks after the first administration, respectively; 16 weeks later, EASI 50 was 92.7%, and EASI 75 was 63.6% (22). Real data from Italy showed EASI 50, EASI 75, and EASI 90 of 98.1, 81.5, and 50.8%, respectively, after 16 weeks (23). In the Netherlands, 85.7, 61.7, and 24.1% of patients achieved EASI 50, EASI 75, and EASI 90, respectively, after 16 weeks of treatment (24). EASI 50, EASI 75, and EASI 90 of the above countries are summarized in Figure 3. The therapeutic efficacy of dupilumab in our patients was better than that reported in other cohorts. The reason why treatment among our cohort is more effective than other populations may be that we had a larger proportion of patients who used combination drugs, including TCS and/or TCI. Compared with the Netherlands, the better curative effect in our cohort may be due to the higher disease severity in the Dutch cohort, in which 61% of patients failed to use ≥2 kinds of immunosuppressive drugs and with the mean baseline EASI (IQR) as 19.9 (13.6–28.3) higher than 19.5 (11.4–17.1) in our cohort. Our study only included a very small number of patients who had previously used immunosuppressive agents (11.21%) or systemic glucocorticoids (4.31%), although with serious condition.

In our study, older patients and those with a longer disease duration were found to have better efficacy with dupilumab. Similar to the results of an Italian real-world study in which elderly patients (≥65 years) with severe AD treated with dupilumab showed significant and persistent improvement in EASI, Pruritus-NRS, Sleep-NRS, and DLQI at 16 weeks and 52 weeks compared with baseline (25). Identifying this characteristic will lead to more informed drug choices in the future.

**FIGURE 4:**
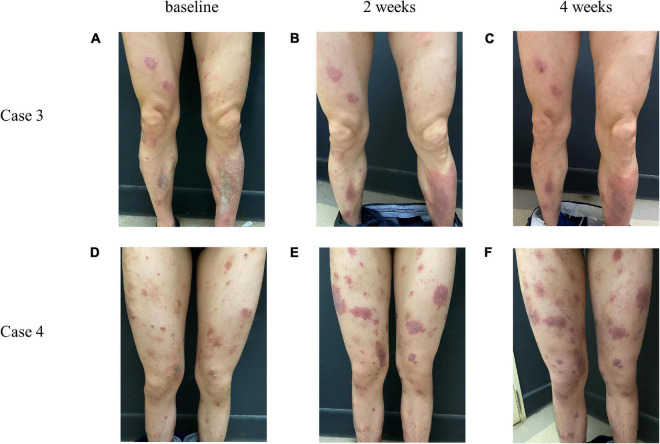
Representative photos of AD patients whose conditions did not improve after treatment with dupilumab. Case 3, case 4 represent two different patients. From left to right, respectively, for the baseline **(A,D)**, 2 weeks **(B,E)**, and 4 weeks **(C,F)** after giving dupilumab.

Eleven patients (9.48%) had a poor curative effect on dupilumab. After dupilumab treatment, one of their disease severity scores occurred an increase compared with baseline. Among them, 6 stopped taking dupilumab at 4 weeks. Eruption on the body was not alleviated and even tended to expand with exudation, and symptoms of itching also persisted. One patient was treated in combination with systemic antibiotics and then alleviated gradually. The characteristics of these eleven patients are summarized in Table 5 and representative photos of these patients are shown in Figure 4.

**TABLE 5:** The characteristics of eleven patients had a poor curative effect on dupilumab.

Characteristics	Mean (±SD) or Count
Gender	Male, 9; Female, 2
Age (year)	35.82 (±19.78)
Course of disease (year)	7.09 (±10.96)
Atopic/allergic diseases at baseline	Yes, 4; No, 11
Baseline serum IgE level (IU/ml) (*N* = 9)	1814.10 (±2572.42)
Baseline eosinophil counts (×10^9^/L) (*N* = 9)	0.81 (±0.80)
Baseline serum LDH level (U/L)	211.00 (±34.40)
Treatment time*	2 weeks, 3; 4 weeks, 6; 6 weeks, 2

*Among eleven patients, 3, 6, 2 stopped taking dupilumab at 2 weeks, 4 weeks, and 6 weeks, respectively.

There may be two reasons for poor efficacy of dupilumab among the 11 patients in our cohort. (1) Secondary infection may be associated with poor efficacy, and AD is accompanied by an increased risk of secondary infection, defects of the skin barrier, inhibition of skin innate immunity by type 2 inflammation, *Staphylococcus aureus* colonization and skin ecological imbalance (26). (2) Although the immunophenotype of all populations is significantly associated with Th2 activation in the pathogenesis of AD, there are some differences. Activation of Th17/Th22 cells in Asian AD patients is stronger than that in American AD patients (27, 28). The poor efficacy of some patients may be because of the significantly high induction of Th17- and Th22-related cytokines.

We observed that treatment of head and face lesions is more difficult than that of trunk and limbs, which is consistent with real-world data reported in Japan (29). Patients with a rash similar to psoriasis (predominantly erythema and scales) also do not respond well to dupilumab, suggesting a possible abnormal Th1/Th17 inflammatory response in these patients.

In our patients, one patient with liver cirrhosis and seven patients with chronic renal failure on regular hemodialysis responded well to dupilumab without side effects, and after two doses, their EASI score decreased by more than 50%. Their skin lesions subsided, with mild tingling. Itching caused by chronic kidney disease-associated pruritus (CKD-aP) or jaundice related to liver cirrhosis may be one of the reasons. Potential pruritogens for CKD-aP include toxin deposition, peripheral neuropathy, Th1 cells, and interleukin, among others (30). Bile salt, histamine, serotonin, steroids and others are causes of jaundice pruritus (31). As dupilumab targets interleukin 4 (IL-4) receptor α, it did not completely relieve itching in these patients. The long-term effect remains to be seen and needs to expand the sample size.

**FIGURE 5:**
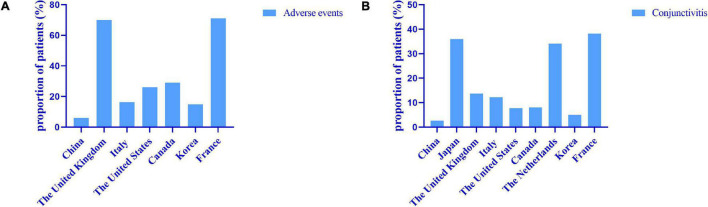
Real world data on adverse events **(A)** and conjunctivitis **(B)** from different countries. The reported rates of adverse events in other countries are higher than those in the Chinese population, especially for conjunctivitis.

Conjunctivitis is the most common adverse reaction. The occurrence of conjunctivitis in our study was 2.59%, and all of these patients had a history of conjunctivitis. In a real-life study in South Korea and Canada, the incidence of conjunctivitis was 4.95% (22) and 7.69% (32), which was similar to our results. In the United States, 27.27% of patients had adverse eye reactions, with conjunctivitis in 7.80%, though how many patients had symptoms of dry eyes and blurred vision was not assessed (33). In Italy, 12.15% of patients developed conjunctivitis, and the risk of conjunctivitis was associated with early onset of AD and eosinophilia (23). Conjunctivitis occurred with a higher ratio of 36–38.2% of patients in Japan and France (34, 35). A clinical trial showed that the occurrence of conjunctivitis is associated with a history of conjunctivitis and elevated baseline IgE or eosinophils and concluded that conjunctivitis is a comorbidity of AD (36). A total of 62.11% of patients in the United Kingdom developed ocular adverse reactions, including 13.68% with conjunctivitis, but the prevalence of pre-existing ocular diseases is unknown; 8.42% of patients developed orofacial herpes simplex, and 4 of them used a combination with immunosuppressive agents, which may be related to 45.26% of patients using immunosuppressive agents in their cohorts (37). In general, the reported rates of adverse reactions in other countries are higher than those in the Chinese population, especially for conjunctivitis (Figure 5), which may be related to racial differences or other unknown reasons.

Limitations of our study include the following three points: The first one is the small sample size due to the fact that dupilumab has only been on the market for more than 1 year. The second one is lack of a control group. The third point is not all patients have used dupilumab for 16 weeks because some of them already got a perfect response and transfer to other traditional treatments or some of them cannot afford it for a complete course of 16 weeks, since dupilumab was not covered by China’s medical insurance during the follow-up period. Further research should investigate on larger sample size and longer follow-up time.

## Conclusion

This real-world study presents outcomes of a cohort of Chinese patients with AD who used dupilumab. The follow-up period was from the start of dupilumab to the end of 16 weeks. The patient characteristics were comparable with those reported in clinical trials and other real-world studies (14, 15, 38). Overall, SCORAD index, EASI, POEM, DLQI, and NRS scores decreased significantly after treatment. The curative efficacy of dupilumab at 4th weeks was related to the patient’s age and course of disease. To our knowledge, this is the first report of the therapeutic efficacy and side effects of dupilumab in controlling symptoms and signs of AD in a Chinese population, and our findings provide a practical basis for future research on dupilumab.

## Data Availability Statement

The original contributions presented in the study are included in the article/supplementary material, further inquiries can be directed to the corresponding author.

## Ethics Statement

The studies involving human participants were reviewed and approved by the Medical Ethics Committee of Xiangya Hospital, Central South University. Written informed consent to participate in this study was provided by the participants or their legal guardian/next of kin. Written informed consent was obtained from the individual(s) or their legal guardian/next of kin for the publication of any potentially identifiable images or data included in this article.

## Author Contributions

BZ and CP wrote the first draft of the report. LL, RL, and LZ revised and reviewed the manuscript. LL, RL, LZ, XC, and JL supervised the study. All authors contributed to interpretation of results and revised the final manuscript.

## Conflict of Interest

The authors declare that the research was conducted in the absence of any commercial or financial relationships that could be construed as a potential conflict of interest.

## Publisher’s Note

All claims expressed in this article are solely those of the authors and do not necessarily represent those of their affiliated organizations, or those of the publisher, the editors and the reviewers. Any product that may be evaluated in this article, or claim that may be made by its manufacturer, is not guaranteed or endorsed by the publisher.
